# THERACOM: a systematic review of the evidence base for interventions to improve *Therapeutic Communications* between black and minority ethnic populations and staff in specialist mental health services

**DOI:** 10.1186/2046-4053-2-15

**Published:** 2013-02-25

**Authors:** Kamaldeep Bhui, Rosemarie McCabe, Scott Weich, Swaran Singh, Mark Johnson, Ala Szczepura

**Affiliations:** 1 Barts and the London School of Medicine & Dentistry, Wolfson Institute of Preventive Medicine, Room Number: OAB 108, Centre for Psychiatry, Old Anatomy Building, Charterhouse Square, 6BQ, London EC1M, UK; 2 Social and Community Psychiatry, Newham Centre for Mental Health, Cherry Tree Way, Glen Road, 8SP, London E13, UK; 3 Mental Health & Wellbeing, University of Warwick, Medical School Building, Gibbet Hill Campus, CV4 7AL, Coventry, UK; 4 MSRC/CEEHD, De Montfort University, Hawthorn Building 00.20, The Gateway, LE1 9BH, Leicester, UK; 5 Health Sciences, Warwick Medical School, Social Studies Building, Main Campus, CV4 7AL, Coventry, UK

**Keywords:** Interventions, Therapeutic communications, Black and minority ethnic patients, Psychiatric services

## Abstract

**Background:**

Black and Minority Ethnic (BME) groups in receipt of specialist mental health care have reported higher rates of detention under the mental health act, less use of psychological therapies, and more dissatisfaction. Although many explanations have been put forward to explain this, a failure of therapeutic communications may explain poorer satisfaction, disengagement from services and ethnic variations in access to less coercive care. Interventions that improve therapeutic communications may offer new approaches to tackle ethnic inequalities in experiences and outcomes.

**Methods:**

The THERACOM project is an HTA-funded evidence synthesis review of interventions to improve therapeutic communications between black and minority ethnic patients in contact with specialist mental health services and staff providing those services. This article sets out the protocol methods for a necessarily broad review topic, including appropriate search strategies, dilemmas for classifying different types of therapeutic communications and expectations of the types of interventions to improve them. The review methods will accommodate unexpected types of study and interventions. The findings will be reported in 2013, including a synthesis of the quantitative and grey literature.

**Discussion:**

A particular methodological challenge is to identify and rate the quality of many different study types, for example, randomised controlled trials, observational quantitative studies, qualitative studies and case studies, which comprise the full range of hierarchies of evidence. We discuss the preliminary methodological challenges and some solutions. (PROSPERO registration number: CRD42011001661).

## Background

### Background policy and research

The challenges faced by people from a black or minority ethnic group when they come into contact with psychiatric services are well documented in previous research reviews and in evidence-based policies [1,2]. These highlight ethnic inequalities of experiences and outcomes, including concerns about patient safety, disproportionate number of admissions and detentions in psychiatric hospitals, conflict with carers and staff, fear of services, lack of engagement or poor access to effective services, anxieties about contact with the criminal justice system and police, a lack of available psychological therapies and inequalities in pharmacotherapy.

### Culture and communication

Clearly, the ability to communicate effectively and in a culturally appropriate manner underpins successful diagnosis and therapy. For example, linguistic isolation at the time of illness can lead to further anxiety and uncertainty in communication during assessment, diagnostic practice and clinical decision-making. Inappropriate use of family or friends as an interpreter to address this issue may still undermine precise assessment; use of bilingual professionals or interpreters with special expertise in mental health settings can improve this [3-5]. However, dissatisfaction and inequalities are also prominent among Anglophone migrants and other people from BME groups who speak English [4,5].

Therefore, the causes of dissatisfaction with care, failure to engage with services or accept treatment, and fears about safety may be explained by inherent communication problems that reflect different underlying assumptions and expectations about the causes and treatments of mental and emotional distress [6]. Ineffective communication and failed negotiation because of these differences may then lead to a feeling of not being understood, omissions of important information from the clinical assessment, conflict with staff, disengagement and/or a failure to take up interventions [6,7]. This may lead to more severe and more frequent episodes of illness and in turn the use of coercion, which is also associated with a higher rate of adverse incidents. Such a cycle undermines the therapeutic potential of existing care practices and processes, but may also add additional burdens on the mental health of service users. Thus, improving therapeutic communications may permit maximum benefits to be realised from existing care and services, improve safety and avoid adverse incidents in care.

Effective communication is central to psychiatric assessment, diagnosis, engagement and treatment, and ultimately recovery [6,7]. Effective communication has proven more difficult to achieve where there are differences in culture or language between those delivering and receiving care [6]. Of course, communication difficulties might also arise from any encounter between a patient and professional because of differences in age, gender, social status or perceived power status. However, cultural differences between patient and professional add additional challenges, for example, the ability of the professional to:

•identify with and empathise with a patient from a different culture [8,9]

•understand symbolic and metaphorical language that varies by culture [10]

•understand differing expectations of health care professionals in different countries and cultures (e.g. authoritarian versus egalitarian approaches, medication as treatment rather than discussing emotional issues) [11];

•appreciate the differences in illness perceptions and explanatory models of patients from different cultures [6].

Cultural factors amplify the limitations of therapeutic communications and are of importance given the potential to compound inequalities in the social determinants of illness and to perpetuate inequalities in health care outcomes following contact with health systems [11-13]. Therapeutic communication can be central to reducing inequalities. For example, Lorenz and Chilingerian, using visual supports for communication, have recently argued that these help address inequalities and gender disadvantage by introducing a more ‘fair process’ of assessment [14]. They define a fair process as one that involves patients in a collaborative approach to explore diagnostic issues and treatments, explains the rationale for decisions, sets expectations about roles and responsibilities, and implements a core plan and ongoing evaluation. Fair process opens the door to bringing patient expertise into the clinical setting and the work of developing health care goals and strategies. Although improved therapeutic communication is at the heart of this fair process, the evidence base to support professionals in achieving this is currently scattered across a number of disciplines and based on different theoretical models. There is a therefore a need to pull this evidence together and appraise its quality in the main areas highlighted in the research brief.

### Cultural competency

One proposed solution has been the dissemination of ‘cultural competency’ training [15]. A review of the international literature on cultural competency suggests that it is best conceptualised as a systemic and deep-seated process of change in both organisations and professional practice [16]. This requires a change in the attitudes of staff and a change in the way they assess, diagnose and treat people with different expectations and perceptions about what is illness and what is recovery. At an organisational level, changes required include developing values that are more welcoming of culturally diverse populations and changes in management styles and HR practices that reflect an understanding of the influence of culture on communication. Alongside these macro-level interventions, educational solutions have been proposed including training to address individual staff attitudes and stereotypes, in order to permit staff to work more effectively with culturally diverse populations. However, the complex introduction of change at an individual and organisational level, linked by changing values and attitudes, has not been widely applied in the UK. Short-term educational solutions have been more popular and therefore more widely reported in the literature. These have varied in quality and focus, with some attending to communication, some to clinical skills and practices, some to the attitudes of practitioners and their cultural biases, and some to specific groups such as faith groups, refugees, migrants, gypsies, or racialised groups. This has made the development of a robust evidence base problematic.

Some cultural competency training has included information on race equality and recruitment legislation mainly to ensure compliance. The Department of Health rolled out a race equality and cultural competency framework to address stigma, race equality and cultural factors [17]. This attempted to present communication issues and sensitivity to stereotypes according to race and culture, but included a limited focus on clinical assessment, diagnosis or specific treatment strategies. Bennett et al. mapped cultural competency training and its content in the UK and concluded there was insufficient attention to clinical interventions and to racial issues, suggesting instead that non-therapeutic communication issues were more prominent in the literature [15]. A systematic review of the international literature on cultural competency interventions in mental health settings has similarly identified few evaluations, and none with patient reported outcomes [16]. A systematic review of therapeutic communications is necessary to synthesise the findings across these many approaches and identify lessons for policy, practice and research.

### Narratives, ethnography and diagnosis

The meaning a person assigns to an illness may be quite different from the formulation of the health professional [18]. This issue is not confined to the UK and reflects fundamental differences across national, cultural, ethnic and religious groups in the way mental distress and illness is understood and defined, and related to expectations of recovery and treatment [19,20]. Canales et al. describe ‘narrative interaction’, sharing of personal stories, as a form of therapeutic communication that permits the gendering of inequalities to be addressed in nursing practice [12].

Making a more detailed assessment of patients’ illness models is advocated by some medical anthropologists; for example, ‘mini-ethnography’ has been used in the clinical assessment in cultural consultation [9]. Studies of cultural consultation have demonstrated improvements in diagnostic precision, diagnostic depth and care plans. Attempts to introduce ethnography in the diagnostic process have led to support for a ‘cultural formulation’, which is highlighted in the diagnostic and statistical manual (DSM-IV, 4th edition) [21]. This advocates that assessment includes ethnography and narrative by asking questions about cultural identity and explanatory models. Explanatory models in the anthropology literature are similar to illness perceptions reported in the psychology literature, and both refer to concepts about what causes illness, what it is called, who might help in recovery and what expectations there are of potential carers. In addition the cultural formulation also asks about psychosocial factors and brings the clinician’s perspective into play by openly seeking comment on interpersonal interactions before seeking an overall judgement about diagnosis and formulation. Although cultural formulation has been reported to be helpful in clinical practice, the published literature mainly contains qualitative and descriptive papers, including case reports; evaluative studies may only appear in the grey literature. Other developments in the UK include a conflict resolution and mediation approach pioneered by Kilshaw et al. [22] and a cultural consultation service that is collecting pilot data on workforce development, cultural competency and organisational narratives of care and communications; the data will show if these influence care practices [22,23]. At the heart of these approaches, ethnography, patient narratives and negotiations of meaning seem to be the key ingredients that benefit patients in these pioneering services [24].

## Methods

### Aims and objectives

We shall conduct a systematic review of the research evidence on interventions to improve ‘therapeutic communication’ among black, minority and ethnic (BME) patients receiving specialist psychiatric care and the professionals who deliver that care.

Within this overall aim, our specific objectives are:

(1) To review the published evidence as well as unpublished ‘grey’ literature and unreported research in order to identify promising interventions to improve ‘therapeutic communication’ for BME patients receiving specialist psychiatric care. Our initial analysis has identified that interventions of interest can broadly be defined as those that:

(a) aim to improve outcomes from existing care through mediation, better understanding and take up (for example, by psycho-education that enhances communication);

(a) seek to manage divergent views, conflict and differing explanatory models and illness perceptions through negotiation and mediation;

(a) employ cultural consultation models and other narrative based or ethnographic methodologies;

(a) involve methods proposed within the social sciences or communications studies, for example, linguistics, but applied to health and social care;

(a) apply cultural competence interventions that aim to improve communication;

(a) improve two-way communication as a therapeutic tool through technology (e.g. NHS direct, telemedicine, email).

2) To report evidence on effectiveness, quality and cost-effectiveness using measures of patient reported outcomes, symptoms, (dis)engagement with care, cost, safety, rates of adverse incidents (including the use of compulsion such as sectioning or physical restraint) and/or use of other interventions (including medication).

3) To identify and describe the elements of identified interventions.

4) To produce recommendations for practitioners and policy makers for different service contexts, patient groups and illnesses.

5) To identify key evidence gaps and highlight future primary research required to address these.

### Review overview

The review will be carried out through a systematic examination of the relevant literature. In so far as is possible, it will conform to the methods and standards of the Cochrane Collaboration [25] but permit review of this protocol where appropriate [26]. It is likely that we will be reviewing a broad range of study designs so a slightly more flexible approach may be more appropriate. Previous work has also found that many studies for review (up to 50%) in areas such as this are found by snowballing [27]. Thus, we will use the principles advocated by the Cochrane and Campbell Collaborations and adapt these for the much broader range of study designs likely to be of interest in relation to assessing the evidence on interventions seeking to improve therapeutic communication amongst black, minority and ethnic (BME) patients receiving psychiatric care. The aim will be to identify qualitative and quantitative research evidence on promising interventions and the elements that appear most important in contributing to their success. The research team’s experience of systematic reviews of ethnicity and health-related studies, particularly observational and qualitative studies, is a significant strength in undertaking this work. For example, we have completed a systematic review of pathways into care and ethnicity [28-30], personality disorder and ethnicity [31], chronic fatigue and ethnicity [32], communication in health care and implications for ethnic minorities and migrants [5], cultural competence in mental health care [16], a review of involving patients in the planning and development of health care [33], self-harm and ethnicity [34], ethnicity and the mental health act [35], and costs of interpreters to the NHS [36].

A protocol for review, as detailed as possible, will be defined at the outset of the study (see below). This will allow the development of initial search strategies and inclusion criteria, and rules of validating these criteria and indicators of methodological quality [37]. The protocol may be broadened as the project progresses. For example, research quality indicators may need to be expanded and further refined in the course of a review of this nature, not least because of the range of methods that studies may utilise.

The review process will consist of three separate stages: literature search; data extraction; synthesis.

### Review framework

Following discussion, the Steering Group will agree on a series of key search terms to be used in the review together with the time scale over which literature will be reviewed. Key aspects will be identified and used to develop a review framework. The review framework will consider and reach agreement on the following:

#### Interventions

At the outset, we define ‘therapeutic communication’ as any conversation (face-to-face or technology assisted) that is undertaken using a pre-defined model that seeks to improve understanding, engagement and therapeutic outcomes. For communication in health care to be therapeutic, it must involve a relationship and exchange of ideas between a patient and professional helper, be patient centred and engaging in order to influence the patient’s emotional world, and directed by the professional using expertise and skill. Therapeutic communications include all interactions that enable people in distress to resolve conflicts, divergent expectations, traumatic histories and adverse life events, and to overcome distress and also take up offers of help.

In this review we are specifically interested in all interventions seeking to “*improve* therapeutic communication amongst BME patients receiving psychiatric care” (e.g. conflict resolution, cultural consultancy, cultural competence and others as yet undefined). These improvements may be aimed at either individuals or populations. Care may be delivered by psychiatrists, GPs, psychologists, nurses or any other professional as long as it is in specialist psychiatric care as set out in the research brief.

Interventions to promote therapeutic communication will be broadly defined as those that:

•employ mediation to enhance mutual understanding and respect to improve engagement with care;

•seek to manage divergent views, conflict, and differing explanatory models and illness perceptions through negotiation and mediation;

•include narrative-based interventions that place the service user and patient perspectives at the heart of consultation, assessment and treatment

•employ cultural consultation models (e.g. the model originating from McGill, Canada)

•apply cultural competence interventions focussed on communication

•any of these processes delivered face to face or through two-way real-time communication technologies (e.g. NHS direct or other support systems, telemedicine, email)

•any new methodology or process for improving therapeutic communications that is not captured by the above, but is suited for BME populations in psychiatric care, and is identified in the literature meeting inclusion/exclusion criteria.

We specifically will not be reviewing the literature on interventions that are considered *to be* therapeutic communications themselves, such as psychological therapies or music therapies, unless the research evidence focuses on interventions that might *improve* therapeutic communications, specifically for BME patients.

#### Patient populations

We are interested in all studies that can provide evidence on how to improve therapeutic communication with BME psychiatric patients in the setting of specialist psychiatric care. Key populations will include all age groups (young people, adults and the elderly) from ethnic groups known to be particularly prominent in health care settings in the UK (namely people from Indian, Pakistani, Bangladeshi, Sri Lankan, Black Caribbean, Black British, Black African, Irish and Chinese backgrounds). However, if identified, we will include data on other ethnic minorities in the UK, e.g. East Europeans.

Although the international literature on minority groups in other countries may not be directly relevant (e.g. African Americans in the USA), it may contain data on approaches to improve therapeutic communication that offer useful insights. Therefore, rather than excluding literature from other countries or national groups, we will, at a first screen, include literature specifically focussed on minorities and migrants in all countries as long as a paper meets our inclusion criteria.

#### Types of research evidence

We will include the full range of experimental (e.g. randomised controlled trials, controlled clinical trials, controlled before and after studies, interrupted time series, before and after, and pilot intervention studies), epidemiological (e.g. case control, cohort, ecological, descriptive and case series) and qualitative (e.g. interview, focus group and ethnographic) study designs. We are also interested in any underpinning theoretical literature of relevance to the success of particular interventions. In addition, we will map all relevant ongoing research projects.

#### Review outcomes

The review outcomes will include a description of promising interventions to improve therapeutic communications and their components; information on mediators or moderators of effects will be captured.

Measures of effectiveness and efficacy of these interventions will be gathered so that the impacts of interventions can be compared and contrasted, alongside a synthesis of evidence of effectiveness and efficacy. Effectiveness and efficacy might be assessed by patient and staff satisfaction, therapeutic outcome measures using patient reports or symptom-based measures, adherence rates, rates of adverse incident reporting, rates of coercive interventions (e.g. medication, sectioning or restraint), rates of disengagement from care, and measures of inequalities by ethnic group in patient outcomes and experiences. These and additional outcomes will be iteratively gathered during extraction and charted so that studies with different outcomes can be contrasted.

Recommendations will be produced for primary research to address important evidence gaps as well as improve the evidence base on any identified interventions that show promise or significant benefits. Further research might also be directed to understand the mechanisms of effectiveness and efficacy, and suitable designs will be recommended, depending on the existing knowledge base in the literature review.

### Literature searches

We will identify all relevant published peer-reviewed work, grey literature and research in progress. Searches will be conducted at the outset of the review and updated to capture more recent material prior to production of the final report. Literature searches will be conducted by a trained information scientist at the Centre for Evidence in Ethnicity, Health and Diversity (CEEHD), Warwick, and a researcher and Librarian at QMUL.

There will be no restriction on language as long as an English language abstract is available for preliminary assessment. Those articles judged to be potentially relevant will be translated. The team have access to an international network of researchers working in the same area, and translations of the small number of non-English articles will be undertaken through existing research networks and learned societies.

The component academic bases, Queen Mary, University of London, University of Warwick and De Montfort, have special collections of ‘ethnic health’ relevance, either through courses delivered by these universities or because of specialist collections kept within their research centres, given the nature of the research priorities of the universities and colleges. University of London also has specialist collections associated with School of African & Oriental Studies (SOAS), and the King’s Fund Library in London also hosts an ethnic health library. Warwick hosts CEEHD.

#### Published articles

Electronic databases will be searched using an optimal combination of MeSH terms to identify all relevant peer-reviewed literature. Since the relevant literature crosses several disciplinary boundaries, it will be important to conduct searches on a range of general as well as specialist databases. Search terms will be adapted for the various databases as in our previous reviews.

#### Search strategy

The preliminary search strategies will be finalised in 3 months. Additional file 1: Annex A1 provides an example of a search strategy developed for MEDLINE to capture articles referring to BME groups; this strategy has been adapted for use in other databases in which articles are indexed differently. Additional file 1: Annex A2 presents indicative results from a preliminary search based on specific key words. Application of filters reduces the 73,892 articles on general therapeutic communications (e.g. intervention/ethnicity) to 103. There is an even larger number of articles on ‘cultural consultation’ (649,950), although the evidence base is more limited for ‘cultural mediation’ (1,233) or ‘conflict resolution’ (7,089). Once again, application of filters reduces the final number of articles. The preliminary search strategies will be refined with our librarian and information scientist and reviewed to maximise the yield as the review progresses. All searches will be repeated at month 12 to identify any new publications.

#### Databases

The databases to be searched include: MEDLINE, PsychInfo, Embase, ASSIA (applied social science index), Cochrane database of systematic reviews, Campbell Collaboration, ACP Journal Club, Cochrane Central Register of Controlled Trials, Cochrane Methodology Register, Allied and Complementary Medicine, CINHAL, British Nursing Index, Health Management Information Consortium, Social Science Citation Index, SocialCareOnline and NHS Evidence collection on ethnicity and health. We will also search university databases for PhD theses (ProQuest assisted) and MSc theses in specialist centres on ethnicity and health. These databases will be searched from inception to 31 January 2012 (proposed start date), and the entire search strategy adopted will be repeated at 12 months.

#### Grey literature

There is likely to be an important body of relevant information contained within the grey literature, including unpublished reports and papers containing relevant information on interventions For unpublished and grey literature, standard database searches will be replaced by a variety of strategies, including ‘hand-searching’ of more recent (last 10 years) issues of journals on ethnicity and health, and journals on communications and ‘cascade-searching’, and by searching specialist collections at the Centre for Evidence in Ethnicity, Health and Diversity (CEEHD), King’s Fund, NHS library on ethnicity and health, HTA, NICE, Royal College of Psychiatrists and Medical Foundation for the Care of Victims of Torture. Our recent reviews have also successfully used various web-based sources (e.g. Google, NHS Evidence) to identify reports that are not published in terms in conventional research or professional journals.

#### Expert networks

Expert networks will be invited to (1) identify omissions in the searches and put forward candidate papers and (2) volunteer research work that is unpublished or in progress.

The applicants and collaborators are in networks in the UK, EU and beyond. Experts will be sent personal invitations to comment on any omissions and to respond to a call for evidence, unpublished data or reports. Experts will be drawn from the Social Perspective Network, specialist email discussion groups (CLAS in the US, Jiscmail in the UK), World Association of Cultural Psychiatry, World Psychiatric Association (Transcultural Section) and the COST EU network on migration and mental health (MigHealth.Net). Community groups and charities will also be contacted to identify materials in community-based collections.

#### Research in progress

Capturing research in progress will be especially important for areas of rapidly expanding practice and research, for example, telemedicine. We will search for research in progress on the National Research Register (US) and the NHS Research Register (UK), both accessible via the British Library. Ongoing trials will be identified through national websites and by writing to the lead author of recent intervention studies in related areas.

#### Bibliography search

The references of all relevant publications will be reviewed and forward and backward citation tracked.

### Selection of material for inclusion in review

The following three-stage approach will be adopted for filtering the large number of papers and other material identified above.

#### First stage: abstract filtering

First stage selection will be based on an examination of abstracts or executive summaries of all material identified through the various search strategies. The research fellow/information scientist will scan all items. Items will be considered for inclusion in the review if they:

1. provide an English abstract, executive summary or full text account (so that a decision can be made on content) or the title unambiguously demonstrates relevance;

2. mention interventions to improve therapeutic communication in patients receiving psychiatric care; and

3. mention ethnic minority groups.

#### Second stage: article selection

All retained abstracts will be inspected against defined inclusion and exclusion criteria by two team members working independently. Articles will be retrieved if they meet the following criteria. For publications about which there is uncertainty, a full text version will be assessed and then another member of the team will adjudicate. Selection criteria will be validated against a sample of ‘out of scope’ papers.

#### Inclusion criteria

Articles that report evaluations or descriptions of (1) models of therapeutic communication to improve assessment, diagnosis, clinical decision-making, treatment and treatment adherence for BME patients, (2) other aspects of direct communication, e.g. consensual/participatory activities, including participatory aspects of cultural consultation, conflict resolution, cultural competence, consent issues, complaints and grievances, drawing up care plans and crisis plans, (3) indirect tele-consultation services (e.g. NHS Direct, telemedicine, e-mail consultations, etc.).

#### Exclusion criteria

Exclusion criteria are articles that simply report on translation or interpreter use in clinical assessment; service delivery to populations speaking diverse languages and evaluations of actual therapeutic communications (e.g. psychological therapies) rather than interventions that might *improve* therapeutic communications.

#### Selecting ‘A’ and ‘B’ papers

Articles with original data and systematic reviews will be rated as ‘A’ articles; other reviews and commentaries will be rated as ‘B’ papers. The intention is that the full text version of all ‘A’ publications will be systematically extracted and analysed and attract quality scores; the references in ‘A’ and ‘B’ publications will be reviewed and subjected to forward and backward citation tracking.

A publication date will be agreed on to act as a filter for the final review; it is likely this will be a date prior to which publications do not usefully contribute to the review question or current and future NHS care contexts. Earlier papers will only be included if more than one member of the research team identifies a particular article as 'seminal', i.e. a well-cited article that contributes substantively to the review.

#### Third stage: inclusion and exclusion criteria applied to full article

On obtaining the full article, it will be possible to examine whether the criteria listed above have been met properly or whether the abstract gives a misleading impression. In our experience this is often the case when key words appear but the full text shows that there is a less detailed analysis of data than expected. The following exclusion criteria will then be applied by two independent readers to all articles selected at stage 2 above:

•excluded if ethnic minorities or ethnicity ‘mentioned in passing’ and not a significant focus

•excluded if no specific focused on *interventions* to *improve* therapeutic communication in patients receiving psychiatric care

•excluded if not appropriate or not relevant to ethnic minorities in the UK (settings or groups examined)

When examining whether ‘ethnic groups’ are discussed appropriately, papers that use the essentially ‘racialised’ notion of ‘non-white’ will, almost without exception, be ignored as grouping together populations whose cultural and other characteristics render any form of generalisation (other than that they were ‘different’ from the ‘majority’) meaningless.

All items successful at this stage will be entered into a central consolidated Review Bibliography. The entire process will be described in a QUORUM flow diagram [26].

### Data extraction and quality assessment

A customised data extraction form will be developed, piloted and refined, and then used by a scientific reviewer to extract data, placing it in charts for comparison by different characteristics of the studies: publication date, study sample sizes and types of study, intervention type, research methods and findings, and quality score. The extracted data will be checked by a second independent scientific reviewer and we will resolve any disagreements by consensus.

The research papers will be assessed and scored for methodological quality using as a starting point schema already used by the applicants in previous systematic reviews. The quality of a study will be rated by discussion between reviewers; in the case of consensus not being reached, a third reader will become involved and, if necessary, arbitrate. Final rating schemes will be produced by testing an initial scheme in early ratings and using the approach advocated by different review bodies as follows:

•For intervention studies we will use the methods recommended in the Cochrane Handbook for Systematic Reviews of Interventions [38].

•For epidemiological studies we will use the MOOSE guidelines for systematic reviews of observational studies, assessing for bias, confounding, regression, heterogeneity and modelling techniques employed [39].

•For qualitative studies we will use guidance from the Cochrane Qualitative Research Methods Group. This will involve assessing the adequacy of study design, recruitment, data generation, reflexivity and analysis (CASP approach) [40].

•Economic evaluation is not central to the review aims and objectives; nonetheless, where economic information is presented and across several studies, we will use the standard Drummond criteria as applied in our earlier reviews [41].

•For quality of description of BME groups, we will use the criteria developed by the CEEHD and implemented by SCEH [42].

### Analysis and synthesis

#### Analysis

We will set up a bibliographic database onto which all articles included in the final review will be entered. Each article meeting the review criteria will be summarised in an abstract and classified by subject, source, the context of the study, methodological type and quality, and key findings. For other articles key words will be recorded. This will provide an overview that will allow us to build up detailed profiles of individual issues, including the quality of the research evidence available for each area and research gaps. Our main analysis will bring together qualitative and quantitative research evidence on:

1. different types of intervention to improve therapeutic communications among BME patients receiving psychiatric care;

2. different categories, formats or elements of therapeutic communication perhaps revealing mechanisms, moderators and mediators;

3. the strength of evidence on efficacy and effectiveness, segmented by study design: pilot studies, definitive trials, observational studies or narrative/qualitative studies;

4. different populations of BME patients receiving psychiatric care; we wish to be able to identify effective interventions that generalise across BME populations. Analysis will consider the evidence available by ethnic minority group, age and gender.

#### Meta-analyses

For trial data, quantitative analysis, outcome, effect sizes and the statistical comparisons of primary and secondary outcomes will be extracted, alongside any narrative outcome of potential explanations for mechanism of effect or adverse incidents. Bias will be considered in assessing methodological quality. If suitable results are identified, we propose to undertake meta-analysis of trial outcomes and observational study outcomes where the outcomes can be summarised in a similar form to permit pooling of estimates. Funnel plots will help identify publication bias. For dichotomous outcomes, we will calculate individual and pooled statistics as relative risks (RR) with 95% confidence intervals. For continuous data, individual and pooled statistics will be calculated as mean differences or standardised means differences with 95% CI. Several packages permit this to be done relatively easily and inexpensively (RevMan, Comprehensive Meta-Analysis, Stata). The research team are experienced at using these packages and providing systematic reviews with meta-analytic outcomes. We may also need to contact original authors of publications if the data are in a form whereby the outcome cannot be easily discerned or is in a form that does not easily permit pooling. We will seek the necessary summary data in the right form for pooling in meta-analysis, subject to ethical permissions and data protection guidance of the original study protocols.

Economic data will be extracted and classified in terms of the economic perspective (hospital, wider healthcare, health and social care, societal) and the type of evaluation undertaken (cost analysis, cost-minimisation analysis, cost-effectiveness analysis, cost-utility analysis or cost-benefit analysis). Historically, we have found better quality economic evidence in grey literature than in peer-reviewed publications [43]. However, should there be sufficient data, analysis and interpretation will be by experts and colleagues in our existing university departments, for example, Health Economics at Warwick, and economic expertise in the Pragmatic Trials Unit at the Wolfson Institute of Preventive Medicine at Queen Mary University of London.

#### Synthesis

Synthesis is a critical part of the review process, involving a critical analysis of the information extracted from the literature reviewed. Synthesis of the information generated in this review will be of supreme importance since policy-makers and others who will need the findings may not be trained in the techniques encountered or in the interpretation of the findings. The main purpose of synthesis will be to provide knowledge relevant to researchers, practitioners and policy makers.

In the synthesis, we will make no attempt to force the finding into an artificial unified framework of analysis, as it is often difficult to combine the results of different types of studies. Instead we aim to use the method of non-quantitative synthesis [which involves literature review, expert reviews of draft material, revision(s) of the draft, and development of policy options or recommendations] used by the US Office of Technology Assessment and by such organisations as the Dutch Health Council. Considerable information can be gained from such qualitative overviews through highlighting variations in the nature and strength of evidence. However, we consider that a meta-narrative approach is also important for research, practice and policy users. Therefore, a meta-narrative review [27], a type of ‘systematic’ review rather than a traditional expert-driven literature review, will ensure that rigorous, explicit and novel conclusions can be credibly drawn from the literature. This is a systematic way to synthesise diverse types of literature with a focus on identifying the ‘storylines of research’ within and across disciplinary boundaries. It will enable us to identify the meta-narratives of each discipline and to analyse the different ‘discourses’.

### Service users and public involvement

Catch-A-Fiya is a network of BME mental health service users, some with skills in research, some in policy and some experts by experience. Catch-A-Fiya will be involved in attending project management and scientific steering group meetings, commenting on methodological challenges and the findings as they emerge, and the interpretation of the overall findings. The network will also help by taking part in a call for evidence; this may be an especially useful way of identifying grey literature and expertise in the voluntary and charity sectors. Members of the network will be able to contribute expertise by reading and commenting on short briefings on the findings sent to them for wider dissemination, ultimately feeding into dissemination. Afiya Trust is a national public charity campaigning for better health among racialised groups; it works in health and social care settings, policy and health promotion; it is a strategic partner of DH that helps building capacity in the charitable sector for inequalities work. This will provide community channels for dissemination as well as the conventional ones through conferences and academic routes, publications in the academic press and in the lay and voluntary sector press. A report launch will be held under the auspices of Afiya Trust in partnership with Warwick, Queen Mary University of London and De Montfort University.

## Discussion

### Key findings

The review will provide meta-analytic, meta-narrative and narrative synthesis of the research literature. The review synthesis will enable robust identification of effective and comprehensive strategies for improvement of therapeutic communications in BME groups across a broad range of study designs, with careful interpretation of the findings [44]. Priorities for future research will be identified through gaps in the evidence base, and also indications of where the evidence is promising and future replication studies or studies of mediation or moderation would be valuable. Our findings will have input from service users from Catch-A-Fiya (a service user network) and the Afiya Trust (a major national BME led third sector organisation), including interpretation of findings and exploring implications for practice, policy and research. This critical stage is often overlooked as a source of potential bias, but recent studies have shown that very different conclusions might be drawn even by experienced researchers [42]. The review will produce recommendations for practitioners and policy makers where the evidence is sufficiently robust, taking account of different service contexts and illnesses. This approach will enable us to provide evidence of practical and policy relevance to inform further actions. This will also highlight research gaps and identify the most promising areas for future primary research.

### Wider context

Interventions may be relevant for improved therapeutic communication in other settings and therefore transferrable; the review will be sensitive to implications for other areas of health and social care (for example, bereavement services, or post-natal depression services or maternity care, and children’s care services). Although such wider concerns are not part of the focussed research brief in this call, we will be able to provide a summary map of the types of evidence discovered in this review of relevance to other areas. This will be a brief non-systematic catalogue only so as not to undermine the key objectives and to preserve resources for the main project. The decisions around which groups to include will reflect relevance to the UK and whether there are lessons for services and interventions in the UK, for example, we will not include components of interventions where there are no evaluations, but primarily we will focus on interventions for which there are evaluations, or in the instance of the grey literature and case studies, where there is an evaluative conclusion.

## Competing interests

Financial competing interests: none.

Non-financial competing interests: Prof. Bhui is Director of MSc Transcultural Mental Healthcare and Director of Cultural Consultation Service at the Wolfson Institute of Preventive Medicine, Queen Mary University of London.

## Authors’ contributions

KB is PI on this HTA-funded project and prepared consecutive drafts of the original protocol with comments and suggestions from the rest of the research team. This article is a shortened and edited version of the original protocol and has not been substantially altered but restricted to protocol information rather than information sought by a funding agency. KB led on the re-edit of the original protocol; otherwise all authors contributed. All authors are contributing to the review through the steering group; KB is overall lead and leads specifically on the published quantitative and qualitative literature synthesis; MJ and AS lead on the grey literature and economic analyses. All authors were either applicants or collaborators on the original proposal. All authors read and approved the final manuscript.

## Authors’ information

The project will be led by Prof. Kamaldeep Bhui at QMUL, applicants at QMUL (Dr. R. McCabe), Warwick University (Prof. S. Weich, Prof. S.Singh) and collaborators at Warwick and De Montfort Universities (Prof. A. Szczepura, Prof. M. Johnson) and at Afiya Trust (Rampaul Chamba and Sola Afuape). Afiya and its related service user networks will be the main avenue of public-patient involvement through its national networks. The team are experienced in mixed methods research, systematic reviews and evidence synthesis across a broad range of study designs. The team are also experienced in developing policy, implementing and testing policy, health services research and clinical practice issues. The team members are part of an effective collaborative group and associated networks, and are all committed to quality in scientific research and evidence synthesis.

**Prof. Kamaldeep Bhui** is a psychiatric epidemiologist and has expertise in systematic review methods for randomised trials and meta-analysis, observational studies and synthesis of grey literature; published systematic reviews include review of ethnicity and outcomes of detention and admissions, ethnicity and self harm and suicide, ethnicity and personality disorder, work stress, ethnicity and chronic fatigue, and cultural competency. KB is developing a cultural consultation intervention that is gathering pilot data; he has been part of a national evaluation of Delivering Race Equality and undertaken population studies of adolescent and adult mental health and health services research including ethnicity and pathways into care (EPIC). KB led the team preparing the compendium of mental health outcomes and chaired the Access & Engagement sub-panel of the NICE guideline update on schizophrenia. He is a member of the EU and International networks on migration and mental health, ethnicity and health, and cultural psychiatry; he works with community agencies and charities promoting health and well-being in populations and in specialist psychiatric care.

**Dr. Rose McCabe** has expertise in mixed methods studies and systematic reviews and a wide knowledge of methods used to analyse communication in psychiatry. Her research focuses on identifying effective communication and improving communication to optimise the experience of treatment and patient outcome. Relevant current projects include studies of how suicide risk assessment is conducted, communication in treatment and its association with patient outcome and communication skills training to improve communication about psychosis.

**Prof. Scott Weich** is a psychiatric epidemiologist with a track record of research into determinants and outcomes of mental disorders. His research includes investigation of all forms of inequality in rates of the most common mental disorders. He has collaborated on several national surveys of psychiatric morbidity in the UK, including EMPIRIC – the largest study of mental disorder in ethnic minorities in the UK. He was lead investigator on a qualitative study of the experiences of users of acute mental health services in a deprived, multi-ethnic inner city community and (with KB and SS) on the evaluation of the national Focused Implementation Site (FIS) roll-out – a key element of the Delivering Race Equality (DRE) programme.

**Prof. Swaran Singh** is an expert in early intervention services for people with schizophrenia and completed a systematic review of the mental health act and ethnicity. He has studied transitions from child to adult services and holds an NIHR programme grant on ethnicity and mental health, and pathways to care.

**Prof. Ala Szczepura** is Professor of Health Services Research at Warwick Medical School. She has over 20 years of experience in policy and evaluative research in health care. She has a long-standing interest in provision of services to meet the needs of diverse populations. In 2001 she was awarded a grant to establish a UK Centre for Evidence in Ethnicity, Health and Diversity (CEEHD) jointly at Warwick and De Montfort University as part of the ESRC’s evidence-based policy and practice initiative. In 2004, CEEHD was appointed to develop a Specialist Library for Ethnicity and Health for the NHS Knowledge Management Programme. In 2009, the Collection became part of a new initiative NHS Evidence under the direction of NICE. She has experience of undertaking a large number of reviews in the area of ethnicity and health.

**Prof. Mark Johnson** has 30 years of experience in conducting research into ethnicity and health issues and specialises in community/user-linked research approaches. He has also worked extensively with Prof. Szczepura and others in the conduct and analysis of structured reviews that include and critically assess the contribution of 'grey literature' and community-based studies. He is the Specialist Advisor on ethnicity and equality issues for NHS Evidence (NICE) and will also manage the linkages with Afiya Trust and other community-based user perspectives as well as the 'research user' community of practitioners (including the 560-strong membership of the electronic community of practice), which will be used to locate work in progress and unpublished studies. He will assist in the location, grading and evaluation of unconventional sources of evidence.

## Supplementary Material

Additional file 1 **Annex A1.** Provisional search strategy for MEDLINE for capturing diverse ethnic groups. Annex2: preliminary searches on key words using PubMed. Click here for file
