# Supplementary material for: THERACOM: a systematic review of the evidence base for interventions to improve Therapeutic Communications between black and minority ethnic populations and staff in specialist mental health services
Source: Syst Rev. 2013 Feb 25;2:15. doi: 10.1186/2046-4053-2-15 (PMC3599664; doi:10.1186/2046-4053-2-15)
Supplement: Additional file 1 — Annex A1. Provisional search strategy for MEDLINE for capturing diverse ethnic groups. Annex2: preliminary searches on key words using PubMed. [file 2046-4053-2-15-S1.doc]

**Annex A1. Provisional search strategy for MEDLINE for capturing diverse ethnic groups**

| **No.** | **Search history** |
| --- | --- |
| 1 | (multicultural or multi-cultural).mp. [mp = title, original title, abstract, name of substance word, subject heading word] |
| 2 | (crosscultural or cross-cultural).mp. [mp = title, original title, abstract, name of substance word, subject heading word] |
| 3 | (transcultural or trans-cultural).mp. [mp = title, original title, abstract, name of substance word, subject heading word] |
| 4 | (multiethnic or multi-ethnic).mp. [mp = title, original title, abstract, name of substance word, subject heading word] |
| 5 | (multiracial or multi-racial).mp. [mp = title, original title, abstract, name of substance word, subject heading word] |
| 6 | (migrant$ or immigrant$).mp. [mp = title, original title, abstract, name of substance word, subject heading word] |
| 7 | refugee$.mp. [mp = title, original title, abstract, name of substance word, subject heading word] |
| 8 | cultural diversity.mp. [mp = title, original title, abstract, name of substance word, subject heading word] |
| 9 | (multilingual or multi-lingual).mp. [mp = title, original title, abstract, name of substance word, subject heading word] |
| 10 | (romany or romanies or gypsy or gypsies).mp. [mp = title, original title, abstract, name of substance word, subject heading word] |
| 11 | asylum seeker$.mp. [mp = title, original title, abstract, name of substance word, subject heading word] |
| 12 | (arab$ or somali$ or yemini$ or Vietnamese or chinese or caribbean or pakistani$ or indian$ or bangladeshi$).mp. [mp = title, original title, abstract, name of substance word, subject heading word] |
| 13 | (Islam$ or Hindu$ or Sikh$ or buddhis$ or muslim$ or moslem$).mp. [mp = title, original title, abstract, name of substance word, subject heading word] |
| 14 | mixed race$.mp. [mp = title, original title, abstract, name of substance word, subject heading word] |
| 15 | (ethnocultural or sociocultural).mp. [mp = title, original title, abstract, name of substance word, subject heading word] |
| 16 | diverse population$.mp. [mp = title, original title, abstract, name of substance word, subject heading word] |
| 17 | ((Black or ethnic or minorit$) adj5 population$).ab,ti. |
| 18 | (BME and ethnic$).ab,ti. |
| 19 | BME.mp. [mp = title, original title, abstract, name of substance word, subject heading word] |
| 20 | or/1-19 |

**Annex2: preliminary searches on key words using PubMed**

|  | Search | Hits |  |
| --- | --- | --- | --- |
| 1 | Interventions | 171,296 |  |
| 2 | Therapeutic communications | 73,892 |  |
| 3 | 1 AND 2 | 3,222 |  |
|  | 1 AND 2 AND ethnic | 103 |  |
| 4 | Cultural competence | 4,200 |  |
| 5 | Cultural consultation | 649,950 |  |
| 6 | Cultural mediation | 1,233 |  |
| 7 | Communication | 429,719 |  |
| 8 | 4 and 7 | 1,144 |  |
| 9 | 5 and 7 | 2,519 |  |
| 10 | 6 and 7 | 412 |  |
| 11 | Conflict resolution | 7,089 |  |
| 12 | 11 and 7 | 289 |  |
| 13 | 1, 2 and ethnic | 81 |  |
| 14 | 7, 11 and ethnic | 104 |  |
